# Less is more: Avoiding the LIBS dimensionality curse through judicious feature selection for explosive detection

**DOI:** 10.1038/srep13169

**Published:** 2015-08-19

**Authors:** Ashwin Kumar Myakalwar, Nicolas Spegazzini, Chi Zhang, Siva Kumar Anubham, Ramachandra R. Dasari, Ishan Barman, Manoj Kumar Gundawar

**Affiliations:** 1 Advanced Centre of Research in High Energy Materials, Prof C R Rao Road, University of Hyderabad, Gachibowli, Hyderabad, Telangana, 500046, India; 2 Laser Biomedical Research Center, G. R. Harrison Spectroscopy Laboratory, Massachusetts Institute of Technology, Cambridge, MA 02139, USA; 3 Department of Mechanical Engineering, Johns Hopkins University, Baltimore MD 21218, USA

## Abstract

Despite its intrinsic advantages, translation of laser induced breakdown spectroscopy for material identification has been often impeded by the lack of robustness of developed classification models, often due to the presence of spurious correlations. While a number of classifiers exhibiting high discriminatory power have been reported, efforts in establishing the subset of relevant spectral features that enable a fundamental interpretation of the segmentation capability and avoid the ‘curse of dimensionality’ have been lacking. Using LIBS data acquired from a set of secondary explosives, we investigate judicious feature selection approaches and architect two different chemometrics classifiers –based on feature selection through prerequisite knowledge of the sample composition and genetic algorithm, respectively. While the full spectral input results in classification rate of *ca.*92%, selection of only carbon to hydrogen spectral window results in near identical performance. Importantly, the genetic algorithm-derived classifier shows a statistically significant improvement to *ca.* 94% accuracy for prospective classification, even though the number of features used is an order of magnitude smaller. Our findings demonstrate the impact of rigorous feature selection in LIBS and also hint at the feasibility of using a discrete filter based detector thereby enabling a cheaper and compact system more amenable to field operations.

Spectroscopic data is widely employed to characterize sample composition and to relate function to the compositional information. In particular, optical spectroscopy methods, notably Raman spectroscopy^1^,^2^, laser induced fluorescence (LIF)^3^, infrared (IR) absorption^4^ and laser induced breakdown spectroscopy (LIBS)^5^, has received considerable attention for high-energy materials (HEM) (explosives) detection because of the lack of sample preparation requirements and ability to provide real-time assessment in stand-off mode. While the vibrational spectroscopic approaches provide a wealth of molecular information, LIBS furnishes complementary elemental information and offers higher signal sensitivity due to the intense emission lines. Researchers have exploited the single-shot and facile elemental analysis capability of LIBS in diverse fields ranging from planetary exploration^6^,^7^, archaeology^8^ to pharmaceutical formulation identification^9^, HEM screening^10^,^11^ as well as in other biomedical^12^,^13^,^14^ and polymer applications^15^. The incorporation of statistical models in the analysis of LIBS coupled with the seminal work by Miziolek and co-workers^16^ and stand-off operations at Los Alamos National Laboratory^17^ has further enhanced its attractiveness for evaluation of complex materials of stochastically varying compositions.

Specific to HEM detection, there are two principal research goals: first, to identify a specimen as a HEM and second, to classify the specimen as a specific HEM. Yet, HEMs typically are nitro-rich molecules and exhibit grossly similar spectral profiles with prominent emission lines corresponding to nitrogen, carbon, hydrogen and oxygen. Furthermore, the spectral interference emanating from trace quantities of grease, oils^18^ and biomaterials^19^ as well as the presence of oxygen and nitrogen in the ambient air, and hydrogen and oxygen in the atmospheric moisture impedes the reliable detection of the materials in the field. Consequently, the basic spectroscopic approach of employing atomic (and in cases molecular) intensity ratios for these kinds of materials yield very limited results. Also, the substrate effects for HEM residue detection, which have been detailed by Gottfried *et al.*^20^, exacerbates the segmentation challenges due to the potential alterations to the light emission from the microplasma. Taken together, it is not surprising that non-analyte specific peaks appear in the spectral profile due to the entrainment of air and the (variable) substrate information^21^. Approaches that partially alleviate these issues include noble gas purging and double pulse method. However, purging is not a viable option for on-field application and while the double pulse method shows some improvement in the signal enhancement^22^, purely ambient air peaks (*e.g.* oxygen and nitrogen) cannot be avoided.

In this milieu, the application of multivariate chemometrics methods to LIBS datasets has enabled the extraction of information both amenable to and hidden from human examination and the resultant combination has been successfully utilized for materials’ identification^23^,^24^. The underlying hypothesis is that while assessing the spectral signatures of several specimen shows significant complexity, comparing hundreds of such spectra reveal subtle, yet reproducible, patterns that are valuable for objective sample identification. Various multivariate segmentation methods including principal component analysis (PCA)^16^,^25^, soft independent modeling of class analogy (SIMCA)^21^, partial least squares-discriminant analysis (PLS-DA)^16^,^21^,^23^, artificial neural network (ANN)^26^ and support vector machine (SVM)^27^ have been exploited to analyze the rich LIBS datasets. Concomitant advances in instrumentation, especially the use of echelle spectrographs, have further enabled measurements of high resolution thereby providing larger number of spectral features for classifier development.

However, the massive amount of data collected puts forth a different challenge as far as the development of a robust classifier is concerned. Given the high dimensionality of the LIBS dataset and the relative sample sparsity (stemming from the unavailability of large numbers of HEM samples), the quest for the ‘perfect’ classifier can often produce unwarranted conclusions and lead to apparently functional algorithms that cannot be used prospectively. Boosting the sample per feature ratio by appropriate selection of spectral regions can not only produce classifiers that are more insensitive to outliers and possess higher generalization power but also aid in interpretability by identification of the smallest possible subset of maximally discriminatory features. Recently, considerable effort has been directed towards developing and evaluating different procedures that objectively identify wavelength bands that contribute useful information and/or eliminate regions that contain mostly noise or spurious information^1^,^21^,^28^. In principle, judicious wavelength selection for spectroscopic datasets focusing on marker-specific features could even result in models having a greater predictive ability. Despite these advantages and the suitability of LIBS for feature selection due to its inherently narrow emission peaks, wavelength selection for building LIBS-based segmentation models has received scant attention in the literature.

Here we present two distinct approaches for wavelength selection in LIBS spectra and investigate their relative merits in influencing the classification outcomes for a set of HEM samples. Specifically, we establish segmentation models based on: (A) spectral regions selected on the basis of *a priori* knowledge of the chemical composition of the samples; and (B) spectral regions determined through implementation of genetic algorithm (GA), a powerful search heuristic that mimics the process of natural selection. First, we examine four spectral regions as well as the full spectrum in constructing PLS-DA derived classification models. PLS-DA is used as a representative multivariate segmentation framework, with the understanding that classification techniques for LIBS and vibrational spectra often provide similar levels of accuracy in prediction due to the richness of the spectral data. The spectral regions were based on the presence of the prominent peak of the organics in the following manner: (a) R1 region that combines the carbon and hydrogen peaks, (b) R2 region that consists of the spectral window spanning from the carbon to the CN lines, (c) R3 region covering the prominent organic window from carbon to hydrogen, and (d) the R4 spectral region encompassing the atmospheric window of the oxygen and nitrogen peaks. Based on this categorization, we find that the correct classification rate using only the R3 region is nearly equal to that of the full spectrum, although it uses ~63% of the intensity values recorded at the detector. Evidently, this finding opens the door for using a detector with significantly smaller spectral coverage that also reduces the possibility of overfitting to variable, yet uninformative, regions of the spectra. Even more significantly, we observe that using the probabilistic, non-local GA search process, a spectral subset featuring an order of magnitude less wavelength information than the full spectrum is capable of surpassing the prediction performance of the latter. The approaches described herein are also sufficiently broad and general to address critical applications in disease diagnosis as well as pharmaceutical and forensic analysis. We also envision that rigorous validation of limited wavelength subsets may provide much-needed momentum to the development of discrete filter-based detector units that can fundamentally reduce the cost, complexity and spatial footprint of the echelle spectrograph – intensified CCD combination of present day LIBS platforms.

## Materials and Methods

Experimental studies were undertaken to accomplish the following objectives: (1) discern the relevant discriminatory features (*i.e.* the differential spectral markers) for the HEM samples under consideration; and (2) use the selected features to establish decision algorithms for HEM sample recognition and determine their sensitivity. For the sensitivity analysis, the rate of correct classification (and corresponding misclassification and unclassification) was computed when all classes of the HEM samples were split into training and testsets.

### Experimental section

A detailed description of our LIBS experimental setup was provided in our previous article^29^. Briefly, a frequency-doubled Nd:YAG laser of 7 ns pulse duration was used as the excitation source. The laser beam was focused by a 10 cm plano-convex lens onto the sample to create the plasma. The plasma emission was collected with a collimating lens assembly CC 52 (Ocean optics Inc.) that was then guided through an optical fiber (core diameter: 400 μm) to the spectrograph (ANDOR Mechelle 5000) coupled to an iSTAR DH734 ICCD detector. The samples were subjected to *ca.* 22 mJ of laser energy with the pulse energy output measured using a pyro-electric joule meter (Molectron, EPM 2000, Coherent Inc). The experiments were carried out in ambient air at atmospheric pressure. A gate delay of 1 μs and gate width 2 μs was used for signal acquisition.

A set of HEM samples namelyoctahydro-1,3,5,7-tetranitro-1,3,5,7-tetrazocine (also known as high-molecular-weight RDX (HMX)), nitrotriazalone (NTO), pentaerythritoltetranitrate (PETN), 1,3,5-trinitro-1,3,5-triazacyclohexane (RDX), 2,4,6-trinitrotoluene (TNT) were acquired from the High Energy Materials Research Laboratory (HEMRL), Pune, India. The fine powder material was pressed into 1 cm diameter pellets by a die-hydraulic pressing machine by applying 3–4 tons of pressure. The pelletization operation improves signal reproducibility, as the position of the focal spot is almost unchanged for all the laser pulses arriving at the pellet surface in sharp contrast to a powder specimen. The pellets were pressed to within 91–94% of the theoretical maximum density for each explosive. The samples were placed on a manual X-Y-Z translation stage such that spectra could be collected from different spatial locations in the pellet to estimate measurement variability.

For our present investigations, 472 spectra were acquired from the HEM samples. Specifically, 133 spectra for HMX, 75 for NTO, 60 for PETN, 61 for RDX, and 143 for TNT were included for the ensuing classification analysis. Each spectral profile represents the signal averaged over 3 consecutive pulses. It is worth noting that the acquired spectra were screened for further analysis by subjecting them to a minimum signal-to-noise ratio threshold and performing spectral outlier detection using correlation distance. In order to prevent the deleterious impact of spectral artifacts, chemometric analysis was performed without the application of any additional preprocessing methods.

## Data Analysis

### Variable selection by genetic algorithm

Wavelength selection for spectroscopy is typically tackled through *a priori* knowledge about the composition of the target specimen. However, as alluded to earlier, complex sample matrix as well as the environment contribute substantive signals from interferents that overlap with the principal features of interest. In order to choose a globally optimal feature subset capable of offering maximal and robust discrimination between the considered HEMs, we employed GA. GAs have provided robust solutions across many applications, offer the possibility to converge on optimal solutions swifter than multidimensional raster grid searches, and possess the ability to escape local maxima during the search for an optimal global solution^30^,^31^. GAs are based on ideas developed in evolutionary biology, where different potential solutions are treated as competing individuals in an evolving population. They have been gainfully utilized for spectral feature selection, where the central idea is the manipulation of binary strings (chromosomes) that contain genes which in turn encode experimental factors or variables^32^,^33^.

Here an initially random population of individuals, or “chromosomes,” was generated with certain characteristics. The optimized GA parameters were: population size, 64 chromosomes; window width, 10%; % initial terms, 1%; max number of generations, 100; percent at convergence, 50%; mutation rate 0.005; regression algorithm and # of LV, PLS and 10 LV’s; cross validation, random and three splits for multiple iterations. For our analysis, we selected spectral subsets in increments of 1% of the total population (25699 points at which intensity values were recorded) with a minimum of 1%. The optimal spectral subset for each increment was selected by maximizing the correct classification rate in a cross-validation routine in the training population.

### PLS-DA multivariate analysis

Although it was originally developed as a multivariate regression method, PLS^34^ has been extensively used to solve classification problems by encoding the class membership of the measured samples in the target matrix^35^,^36^. An important feature of this supervised technique is that it is specifically suited to deal with problems in which the number of variables is large (compared to the number of observations) and collinear, two major challenges encountered when LIBS spectra are used. Furthermore, PLS-DA provides a powerful tool for discrimination even when the variability within a class is similar to the inter-class variability by virtue of establishing the maximal separation between each class via fitting one global model to the entire dataset. In PLS-DA, a PLS regression model is calculated that relates the independent variables (spectral data) to a binary response encoded as: {1, 0, 0, 0, 0} for sample belonging to class 1, {0, 1, 0, 0, 0} for sample belonging to class 2, and so on until class 5. Typically, a threshold value based on the Bayesian method^37^, is defined between 0 and 1, and calculated on the basis of the values predicted during the classification process, so an object is assigned to a particular class if its prediction is larger than the threshold value for this class. As in the PLS regression model, the optimal number of latent variables (LVs) retained are chosen before the modeling process and this is determined by using the fractional misclassification error rate in cross validation^38^.

### Sensitivity Test

The sensitivity investigations were performed for the two approaches(region selection based on *a priori* compositional information and GA) by randomly splitting the 472 sample datasets into 282 for training, 95 for tuning and 95 for test, respectively. Wavelength selection was performed on the training set whereas the tuning set was used to determine the unclassification thresholds for the developed classification model. The test set was kept aside for final classification purposes. The size of the test set represents a compromise between opting for a test set size large enough to minimize the standard error in computing the misclassification rate, while maintaining the training and tuning set sizes large enough to construct and optimize a robust classifier. To obtain more representative rates of correct classification, misclassification and unclassification, 100 independent iterations were performed by re-splitting the entire data into training, tuning and test sets. In the following, we describe the procedure for application of each classifier for one iteration where the dataset has already been randomly split into training, tuning and test sets.

Additionally, to determine the number of the LVs we employed a leave-one-out cross-validation procedure in the training set. Also, for predicting the class of a spectrum, we used equally weighted scaled orthogonal and score distances. Here, using the tuning set, we defined an unclassification criterion to prevent the misclassification of potential samples that are distant from the center of the PLS-DA model. Based on the assumption of normal distribution of the distances of the tuning dataset to the center of the PLS-DA model, distance thresholds were established (mean ± 3.5*standard deviation) to unclassify correspondingly distant test samples. In other words, if the membership probability for every class is observed to be <0.3%, the test sample was categorized as “unclassified”.

## Results and Discussion

### Spectral observations

**Figure 1:**
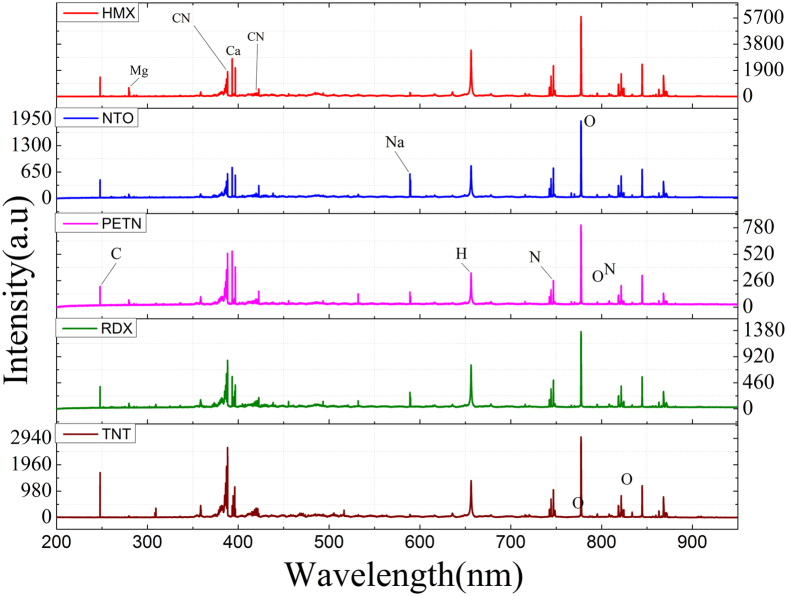
Representative LIBS spectra of high-energy material (HEM) samples: HMX, NTO, PETN, RDX and TNT. The spectra are normalized and offset for visualization purposes.

Figure 1 shows a representative spectrum of each of the five HEM classes measured on our LIBS system. As observed from Fig. 1, the acquired spectra for the 5 classes of HEM samples, namely HMX, NTO, PETN, RDX and TNT, show the presence of emission peaks associated with C (247.8 nm), Mg (279.5 & 280.3 nm), Ca (393.3, 396.8, 422.7 nm), H (656.3 nm), N (742.4, 744.3, 746.9, 818.4, 818.8, 821.6, 824.2 nm), O (777.2, 777.4, 794.8, 822.2, 822.7, 844.6, 868.1 nm) and Na (589, 589.6 nm). Evidently, the oxygen peak at 777.3 nm has the highest intensity followed by CN at 388.3 nm, which along with the C_**2**_ peaks are representative of organic molecules. In addition, the CN violet bands are observed corresponding to B^2^Σ^+^ → X^2^Σ^+^ transitions at three regions with Δυ = −1, 0, and +1 at 357–360 nm, 384–389 nm and 414–423 nm ranges, respectively. C_2_ swan bands corresponding to D^3^Π^g^ → a^3^Π^u^ transitions are also seen in two regions with Δυ = −1 and +1 at 460–475 nm and 550–565 nm ranges, respectively. While the HEM specimen show expectedly near-identical peaks, careful inspection reveals the subtle, but reproducible, peak intensity variations across the samples. The underlying basis of feature selection is to exploit these intensity variations not only at the notable peaks (which often do not provide the highest diagnostic power) but across the spectrum to obtain an accurate classifier and, ideally, identify the smallest spectral subset of discriminatory features.

### Sample compositional knowledge based spectral region selection

The recorded spectra consist of intensity values at 25,699 pixels of the detector, which spans a wide wavelength region from the ultraviolet to near-IR. While the LIBS spectrum consists of several sharp peaks attributed to the elemental (and some molecular) emission lines, a substantial portion of the feature space is composed of minimal signal beyond the noise floor (Fig. 1). However, very rarely is there a concerted effort in choosing the features that is rooted in the context of the application (for example, chemical composition of the samples) – rather for reasons of expediency the full LIBS spectrum is routinely employed in classification. Though inclusion of noise^39^ and continuum^40^ may not significantly hamper the prediction performance in specific cases, feature selection in general is critical to eliminating spurious correlations especially in applications where the inter-class differences are subtle. Here based on the prominent spectral features of CN, C_2_, C, H, O and N, we selected four different regions R1, R2, R3 and R4 as detailed in Table 1. For R1, carbon and hydrogen-specific peaks are included since carbon and hydrogen are the dominant peaks of HEMs and are less influenced by the surrounding ambient atmosphere in comparison with oxygen and nitrogen features. Region R2 is based on the peaks representative of C and CN. The formation of CN depends on the content of carbon and nitrogen in the sample, besides the nitrogen contribution from the atmosphere. Region R3 provides the cumulative information of R1, R2 and C_2_ which is expected to indicate the presence of aromatic double bonds (C = C)^41^. Finally, region R4 consists of the higher wavelength spectral region following the hydrogen peaks and has contributions from both the HEM specimen and the atmosphere.

Using PLS-DA derived classifiers classification performance was determined for each of the spectral regions. Table 2 enumerates the results of correct classification, misclassification and unclassification rates. Here the maximum correct classification rate (92.61%) was obtained when the full spectral profile was considered as the input to the classifier. The misclassification of 4.65% was mainly due to the incorrect recognition of RDX samples that were misclassified as HMX. This misclassification can be attributed to the similar chemical formulation of HMX and RDX and, notably, to the significantly larger number of HMX samples in the dataset that likely ‘skew’ the decision algorithm. The unclassification rate was almost the same for all classes and, as noted from Table 2, for the four spectral regions and the full spectrum. On the other hand, the misclassification rates varied for the selected spectral regions from *ca.* 6.8% for R3 to 28.3% for R1. It is noteworthy that despite the presence of the prominent carbon and hydrogen peaks in R1, its diagnostic power is significantly lower due to the lack of significant variations in this region between the 5 HEM sample classes. Perhaps, more importantly, we observe that there is barely a 2% reduction in the classification performance between R3 and the full spectrum, even though R3 constitutes only ~63% of the full spectrum intensity values. This is not unexpected as the main emission lines that are not incorporated in the R3 come from oxygen and nitrogen – contributions that are marred by atmospheric interference. Evidently, decreasing the size of sampled points not only reduces the redundant and uninformative regions but also raises the possibility of using a spectrometer that is focused only on the lower wavelength region for HEM classification with equal efficacy.

### Unsupervised feature selection using genetic algorithm

**Figure 2:**
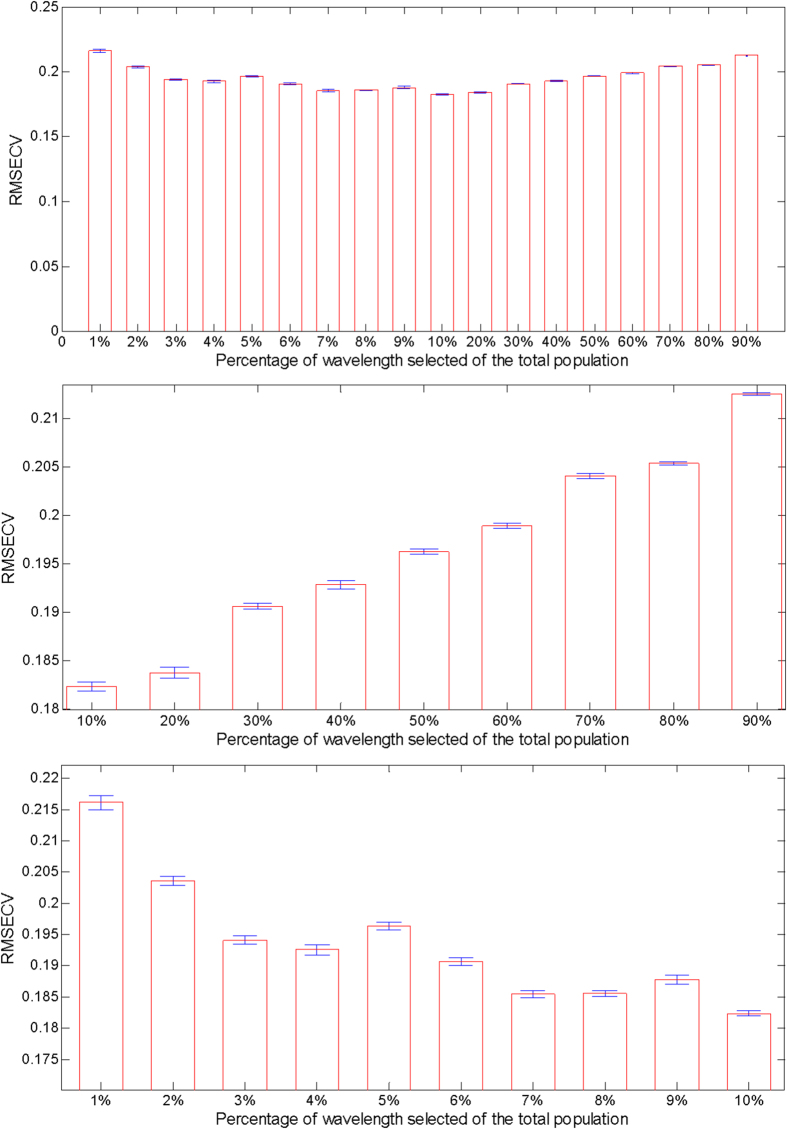
Bar plots with classification error rates showing comparative performance of wavelength-selected PLS-DA classification models. The wavelength selection in this case is performed using genetic algorithm. The length of the bars is proportional to the average root mean square error (RMSE) and the associated error bars represent the standard deviation over a hundred iterations.

While *a priori* knowledge of the sample aids in selection of broad wavelength bands, a more rigorous wavelength selection strategy could potentially yield an even smaller subset of discriminatory features. To this end, we employed genetic algorithm for interrogating the large search space in which extremely diverse combinations of wavelengths must be considered. Figure 2 shows the results of classification for HMX, NTO, PETN, RDX and TNT obtained with the GA/PLS-DA model, where the lengths of the bars are proportional to the average error rate (root mean square error of cross validation, RMSECV) in the training set and the associated error bars represent the standard deviation over 100 iterations. Here RMSEV is computed by comparing the PLS-DA estimates with the assigned numeric values for the reference classes in a quadratic loss function. This provides a direct assessment of the performance of the classification models corresponding to the selection of wavelength subsets, ranging from 1% to 90% of the total population of spectral points (~250 to 23,129 spectral points, where the total population is 25,699). A decreasing monotonic trend is observed for the RMSEV values as the number of points included in the classification analysis is reduced from 90% (0.212) to 10% (0.182) (highlighted in the second panel of Fig. 2). Clearly, this affirms the potential to surpass the prediction performance of full spectral analysis due to the ability to decompose the spectral data matrix in a manner biased toward the isolation of analyte-dependent information – a result that exceeds the findings of the previous approach. At the resolution of our current analysis, 10% of the points sampled appear to provide the optimal balance of selected variables and discriminatory power of the classification model. As the number of wavelengths sampled is further reduced from 10%–1% (0.216) of the total population, we observe a non-monotonic increase in the RMSEV values. Nevertheless, it is notable that selection of only 1% of the wavelengths provides nearly equivalent levels of prediction accuracy (RMSEV = 0.216) as when 90% of the full spectrum is employed. In fact, the corresponding p-value (0.54) indicates the absence of statistically significant differences between the classification performance of the models employing 1% and 90% of the points.

Based on the RMSEV results, one can reasonably infer how many spectral points provide relevant information specific to the analyte of interest and the spectral interferents in the samples – and therefore the minimum size of spectral dataset required for optimal classification performance. Specific to GA implementation, an effective approach for reducing the number of final wavelengths is to reduce the number of wavelengths selected in the initial chromosome^42^. Moreover, with the use of lower spectral resolution, the GA optimization efficiency can be further improved and the number of wavelengths in the optimal chromosome can be further decreased.

Table 3 gives the results of sensitivity analysis for the GA/PLS-DA based classification with 90%, 10% and 1% of the selected wavelengths. The model is judged to be sensitive if the correct classification rate is high and the unclassification rate is low. For the 90% case, we observe a high rate of correct classification for all classes with an average rate of correct classification of *ca.*93%. In particular, no misclassifications are observed for TNT and PETN. However, relatively high rate of misclassification is obtained for the RDX sample that mirrors the findings of the previous region-specific PLS-DA models. Also, from the unclassification perspective, the PETN samples perform relatively poorly in relation to the other HEM classes, which imply the existence of spectral outliers for this class of samples. From the corresponding GA/PLS-DA-based classification with 10% (~2,569 spectral points) of the wavelengths, we find that the average rate of correct classification is even higher (*ca.* 94.2%), with the TNT, NTO and HMX samples being classified with very high accuracy (≥96%). The improvement in the average rate of correct classification from 90% to 10% is statistically significant (*p* < 10^−4^). Additionally, the average rate of unclassification for the 10% case (2.3%) is slightly smaller than in the case of 90% of the selected variables (2.6%) (*p* < 10^−4^). Finally, when only 1% of the wavelengths are included in the analysis framework, we see that the correct rate of classification is slightly lower (90.7%) in relation to the 90% case and, similarly, the average rate of unclassification is also slightly higher (3.2%). The import of this result cannot be understated since nearly same levels of performance are observed while reducing the number of wavelengths used by 2 orders of magnitude (from ~25 k to barely 250). Table S1 (in Supporting Information) also shows the robustness of these wavelength-selected PLS-DA models in correctly identifying and unallocating spectra from samples of an unknown class.

**Figure 3:**
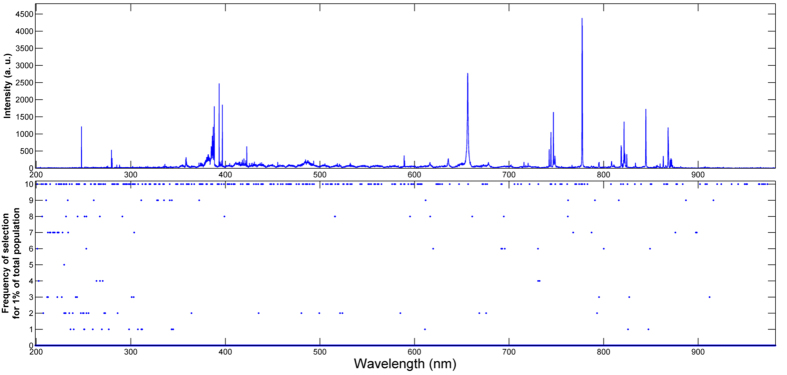
Plot of frequency of wavelength selection for the 1% selected spectral subset over 10 independent iterations with a representative spectrum of HMX on top. 1% of the full spectrum corresponds to 259 spectral points.

Given the significant reduction in the number of sampled points for the 1% case, a critical question that needs to be addressed is the relative consistency of the selected wavelength subset across multiple iterations. Ideally, the selected wavelength subsets should exhibit a high degree of consistency (reproducibility) since these ~250 points are determined based on their discriminatory power - particularly if the selection is not affected by spurious or chance correlations in the models. To quantify the reproducibility, we performed multiple iterations of the 1% case and computed the consistency as the ratio of overlapping spectral points (between selected wavelength subsets across 10 iterations) to the total number of selected spectral points (259). This was found to be equal to 0.72 ± 0.03 that implies over 70% of the wavelengths were common to all the subsets. Figure 3 provides a cumulative frequency plot illustrating the number of times a specific wavelength is retained in the selected subset. The consistency of wavelength selection is represented by the presence of greater structure, *i.e.* higher retention frequency of specific wavelengths and concomitant reduction of other uninformative regions. Figure S1 in Supporting Information shows an alternate representation of the frequency plot highlighting the sampling differences between the 1% and 10% wavelength selected data sets.

Collectively, we have demonstrated the efficacy and consistency of the wavelength selection approach in building classification models for HEM recognition. Despite the limited number of HEM classes used in this research, compared to what may be typically encountered in a defense facility, the variable selection approach by using GA is shown to provide very good compliance and high prediction accuracy in classification of the blind cases.

## Conclusions

Given the large dimensionality of LIBS spectral data, it is critical to develop classifiers that endorse and rely on prior feature selection. Here we have exhibited the efficacy of a supervised as well as genetic algorithm based wavelength selection for discerning the maximally discriminatory features in the spectral data. While our previous work has shown the usefulness of employing multivariate chemometric approaches in segmentation problems using LIBS, this study underscores that the most prominent peaks in the acquired spectroscopic data are often not the parameters with the most diagnostic utility. In particular, our GA-based PLS-DA models demonstrate superior classification performance to corresponding algorithms that employ the full spectral data – highlighting the need of removing uninformative or spurious information that may occupy as much as 99% of the entire information collected. Importantly, we show the robustness (consistency) of the selected wavelength subset regardless of the exact identity of the HEM samples selected in the training set. Through our computational work on feature selection in the sparse LIBS dataset, we also envision architecting a compact, inexpensive diagnostic platform that operates without a conventional echelle spectrograph-ICCD combination while also optimizing the laser excitation wavelength to enable higher pulse energies and improved discrimination. A critical consideration in this regard is the possibility of acquiring lower resolution data and its influence on the quality of the classification model. In combination with our previous report of the better-than-expected performance of non-gated LIBS detection, this report greatly expands the feasibility of field applications while highlighting the untapped opportunities in exploiting computational approaches to make instrumentation innovations and advances. With minimal alterations, the approach described here can be equally exploited in quantitative compositional analysis of pharmaceutical samples, forensic specimen as well as in other recalcitrant process monitoring applications.

## Additional Information

**How to cite this article**: Kumar Myakalwar, A. *et al.* Less is more: Avoiding the LIBS dimensionality curse through judicious feature selection for explosive detection. *Sci. Rep.* **5**, 13169; doi: 10.1038/srep13169 (2015).

## Supplementary Material

Supplementary Information

## Figures and Tables

**Table 1:** Details of selected spectral data regions based on the sample compositional information.

Sl. No.	Region Name	Description	Wavelengths included (nm)	% of full spectrum
1	R1	C and H peaks	246.98–248.05; 640–670	3.16
2	R2	C to CN region	246.98–424.04	34.05
3	R3	C to H region	246.98–670.04	62.85
4	R4	Post-H region	670–875.99	17.06
5	Full	Entire spectrum	199–981	100

**Table 2:** Classification performance of PLS-DA models featuring selected spectral regions.

Region Selected	Correct Classification (%)	Misclassification (%)	Unclassification (%)
**R1**	69.65	28.32	2.02
**R2**	87.48	10.24	2.27
**R3**	90.42	6.78	2.79
**R4**	85.42	12.34	2.23
**Full Spectrum**	92.61	4.65	2.73

**Table 3:** Results of sensitivity analysis for the GA/PLS-DA models for HEM classification.

90% of sampled points	Correct Classification (%)	Misclassification (%)	Unclassification (%)
HMX	95.78	1.93	2.30
NTO	95.87	3.47	0.67
PETN	93.50	0.00	6.50
RDX	81.83	17.00	1.17
TNT	97.66	0.00	2.34
**Average**	**92.93**	**4.48**	**2.59**
**10% of sampled points**	**Correct Classification (%)**	**Misclassification (%)**	**Unclassification (%)**
HMX	96.54	0.80	2.65
NTO	97.67	1.44	0.89
PETN	95.42	1.11	2.92
RDX	83.19	13.75	3.33
TNT	98.16	0.00	1.84
**Average**	**94.20**	**3.42**	**2.33**
**1% of sampled points**	**Correct Classification (%)**	**Misclassification (%)**	**Unclassification (%)**
HMX	93.56	2.67	3.78
NTO	93.07	4.80	2.13
PETN	88.67	5.17	6.17
RDX	79.33	17.83	2.83
TNT	98.62	0.07	1.31
**Average**	**90.65**	**6.11**	**3.24**

Three separate cases are tabulated corresponding to 90%, 10% and 1% of the full spectrum being sampled.
