# Supplementary material for: Less is more: Avoiding the LIBS dimensionality curse through judicious feature selection for explosive detection
Source: Sci Rep. 2015 Aug 19;5:13169. doi: 10.1038/srep13169 (PMC4541340; doi:10.1038/srep13169)
Supplement: Supplementary Information [file srep13169-s1.pdf]

SUPPORTING INFORMATION

Less is more: Avoiding the LIBS dimensionality curse through judicious feature selection for explosive detection

Ashwin Kumar Myakalwar,[‡,ǁ] Nicolas Spegazzini,[†,ǁ] Chi Zhang,[§] Siva Kumar Anubham,[‡] Ramachandra R. Dasari,[†] Ishan Barman,[,§] Manoj Kumar Gundawar[*,‡]*

‡Advanced Centre of Research in High Energy Materials, Prof C R Rao Road, University of Hyderabad, Gachibowli, Hyderabad, Telangana, 500046, India

†Laser Biomedical Research Center, G. R. Harrison Spectroscopy Laboratory, Massachusetts Institute of Technology, Cambridge, MA 02139, USA

§Department of Mechanical Engineering, Johns Hopkins University, Baltimore MD 21218, USA

**Address all correspondence to: G. Manoj Kumar: manoj@uohyd.ac.in; Ishan Barman: ibarman@jhu.edu*

ǁAuthors have made equal contributions to this work

Robustness Test

In the context of explosive detection, an important and realistic scenario consists of taking into account that samples of an unknown class are likely to be encountered. If the identification model behaves correctly, it has to yield that the sample is effectively unknown. This is often referred to as robustness of the classification model. In order to test it, one class was removed from the training set in sequence while the test set remains unchanged. This procedure is alternately repeated for each class. Hence, if the spectra of the unknown test class(that does not appear in the training set)is unclassified by the developed models, the model is considered to be robust (and a positive identification for our statistical analysis). Due to these changes from the previous sensitivity analysis, we have altered the classification categories to the following: “correct allocation”, “misclassification”, and “unallocation”. “Correct allocation” is defined as all correctly classified spectra (from known classes), as well as all correctly unclassified spectra (from unknown classes), whereas “unallocation” refers to unclassification of the spectra from the known classes. Incorrect classification (from known or unknown classes) falls under the general category of “misclassification". This analysis was performed with the 10% spectral subsets established by the genetic algorithm selection process.

Table S1. Robustness Test Results
PLS-DA predictions when samples of a specific class
are alternately omitted from the wavelength selected training set

**Omitted Class**	**Correct allocation**	**Unallocation**	**Misclassification**	**Among allocated spectra, rate of correct allocation**
**HMX**	68.41	26.05	5.54	**92.51**
**NTO**	56.25	26.57	17.18	**76.61**
**PETN**	61.20	27.74	11.06	**84.69**
**RDX**	64.16	28.37	7.47	**89.57**
**TNT**	62.18	30.44	7.38	**89.39**

Table S1 shows that spectra of the unknown sample are effectively unclassified with the average misclassification rate, *ca* 9.7%, being reasonably low. This performance may be further optimized by using other classification strategies such as support vector machines that are known to be more robust to the presence of outliers. Moreover, we note that rates are rather homogeneous and do not strongly depend upon the removed explosive class.

Figure S1

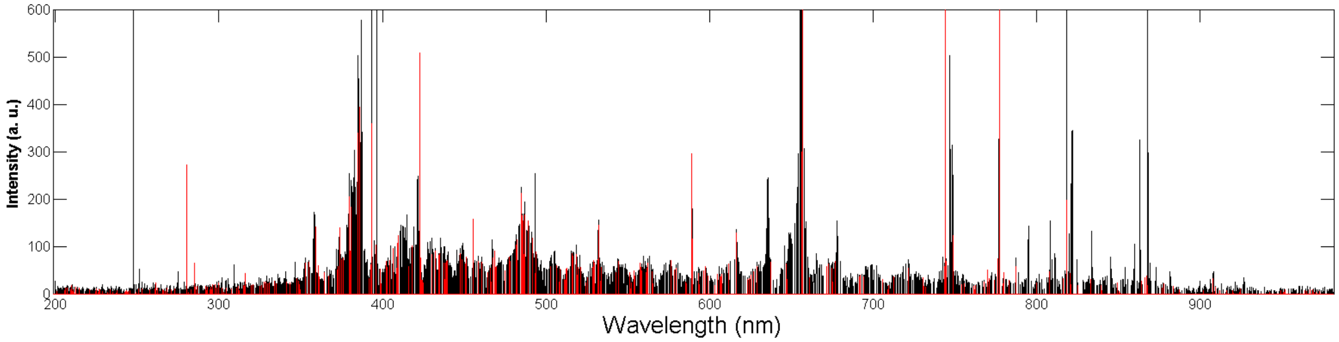

Figure S1:Illustration of the GA-based 1% (red) and 10% (black) wavelength selected features on a representative HMX spectrum. The HMX spectrum is intentionally truncated on the Y-axis to enable better visualization of the lower-intensity emission features selected. This plot reinforces our understanding that the features with the most diagnostic utility may not perfectly correlate with the most prominent peaks in the acquired spectroscopic data. Additionally, we observe that the principal features selected for both the datasets expectedly show a high degree of overlap with the differences stemming from the 10-fold higher sampling frequency of the 10% wavelength set.
